# Chiral Covalent-Organic Framework MDI-β-CD-Modified COF@SiO_2_ Core–Shell Composite for HPLC Enantioseparation

**DOI:** 10.3390/molecules28020662

**Published:** 2023-01-09

**Authors:** Xiaoyan Ran, Ping Guo, Caifang Liu, Yulan Zhu, Cheng Liu, Bangjin Wang, Junhui Zhang, Shengming Xie, Liming Yuan

**Affiliations:** Department of Chemistry, Yunnan Normal University, Kunming 650500, China

**Keywords:** chiral covalent-organic frameworks, core–shell composites, high-performance liquid chromatography, chiral separation

## Abstract

The chiral covalent-organic framework (CCOF) is a new kind of chiral porous material, which has been broadly applied in many fields owing to its high porosity, regular pores, and structural adjustability. However, conventional CCOF particles have the characteristics of irregular morphology and inhomogeneous particle size distribution, which lead to difficulties in fabricating chromatographic columns and high column backpressure when the pure CCOFs particles are directly used as the HPLC stationary phases. Herein, we used an in situ growth strategy to prepare core–shell composite by immobilizing MDI-β-CD-modified COF on the surface of SiO_2_-NH_2_. The synthesized MDI-β-CD-modified COF@SiO_2_ was utilized as a novel chiral stationary phase (CSP) to explore its enantiomeric-separation performance in HPLC. The separation of racemates and positional isomers on MDI-β-CD-modified COF@SiO_2_-packed column (column A) utilizing n-hexane/isopropanol as the mobile phase was investigated. The results demonstrated that column A displayed remarkable separation ability for racemic compounds and positional isomers with good reproducibility and stability. By comparing the MDI-β-CD-modified COF@SiO_2_-packed column (column A) with commercial Chiralpak AD-H column and the previously reported β-CD-COF@SiO_2_-packed column (column B), the chiral recognition ability of column A can be complementary to that of Chiralpak AD-H column and column B. The relative standard deviations (RSDs) of the retention time and peak area for the separation of 1,2-bis(4-fluorophenyl)-2-hydroxyethanone were 0.28% and 0.79%, respectively. Hence, the synthesis of CCOFs@SiO_2_ core–shell composites as the CSPs for chromatographic separation has significant research potential and application prospects.

## 1. Introduction

Covalent organic frameworks (COFs) are multifunctional porous crystal materials formed via the covalent bonding of light elements [1,2,3,4]. These materials have the characteristic of low density, rich active sites, large specific surface areas, and good chemical stability. In the last few years, they have been broadly utilized in many fields, such as drug delivery [5,6], catalysis [7], gas adsorption and separation [8,9], electrochemical sensing [10,11] and chromatographic separation [12,13]. In particular, the usage of CCOFs as novel chiral stationary phases (CSPs) in chromatographic separation has attracted extensive attention [14].

Covalent organic frameworks can be modified by a post-modification strategy because their pores are easy to be functionalized. By covalently connecting the functional groups with the side-chain groups or active sites on the pores of COFs, some new COF materials have been successfully synthesized and used as separation media for chromatographic separation. In 2018, the Cui group [15] reported for the first time that CCOFs with three-dimensional structures were utilized as CSPs for HPLC separation, which has good enantioselectivity for chiral alcohols. Zhang et al. [16] prepared a variety of CCOFs by immobilizing amino acids, lysozyme, and other biomolecules on the achiral COFs to prepare a variety of CCOFs. The prepared lysozyme⊂COF1 showed good enantioselectivity towards various racemates in HPLC. In addition, our group reported that two kinds of CCOFs (CTpPa-1 [17] and CTpTa-2 [18]) simply mixed with C18 silica microspheres were packed into HPLC columns were applied for the resolution of racemic compounds. These studies enrich the construction of novel CCOFs, and expand the prospect of CCOFs as CSPs for chromatographic separation.

As is well known, the research on CCOFs as the HPLC stationary phase is still in its infancy. The prepared COFs crystal particles have the characteristics of irregular morphology, low density, and wide particle size distribution, which will lead to the disadvantages of low column efficiency, undesirable separation performance as well as high column backpressure of CCOFs packed columns. To overcome these problems, silica microspheres with good chemical stability and easy surface modification were used together with chiral COFs to prepare CCOFs@SiO_2_ core–shell composites for HPLC separation. In recent years, many groups have reported the use of COFs@SiO_2_-based core–shell composites for chromatographic separation, such as SiO_2_@rLZU1 [19], TpBD@SiO_2_ [20], BtaMth@SiO_2_ [21], MICOF@SiO_2_ [22], and SiO_2_@COF5 [23]. Furthermore, our group has prepared two chiral core–shell composites (CTpBD@SiO_2_ [24] and β-CD-COF@SiO_2_ [25]) for HPLC enantioseparation. The construction of CCOFs based core-shell composites as CSPs will afford a new stage for the utilization of CCOFs in HPLC.

Cyclodextrins (CD_S_), as chiral selectors, have been widely used in molecular recognition and separation. Because of its easy preparation and suitable cavity size, β-CD is commonly used. Cyclodextrins can be converted into inclusion complexes with organic molecules to produce host–guest interaction [26]. Hence, β-CD and its derivative-based CCOFs have attracted significant attention from researchers.

Herein, a MDI-β-CD-modified COF was prepared with the bottom-up method using MDI-β-CD as the chiral selector obtained through the reaction of β-cyclodextrin and 4,4’-methylenebis (phenyl isocyanate) (MDI). Next, we prepared a MDI-β-CD-modified COF@SiO_2_ composite adopting an in situ growth method and utilized it as a new CSP. The results illustrated that the MDI-β-CD-modified COF@SiO_2_-packed column exhibited good separation ability for positional isomers and racemates, including alcohols, epoxides, ethers, esters, amines and ketones.

## 2. Results and Discussion

### 2.1. Characterization of SiO_2_-NH_2_, MDI-β-CD-Modified COF and MDI-β-CD-Modified COF@SiO_2_

**Figure 1:**
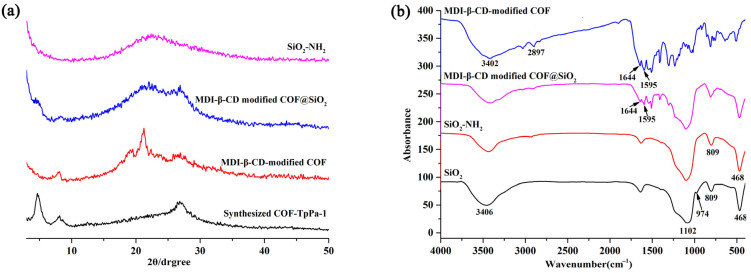
(**a**) PXRD spectra of the synthesized COF-TpPa-1, SiO_2_-NH_2_, MDI-β-CD-modified COF, and MDI-β-CD-modified COF@SiO_2_. (**b**) FT-IR spectra of SiO_2_, SiO_2_-NH_2_, MDI-β-CD-modified COF@SiO_2_, and MDI-β-CD-modified COF.

The SiO_2_-NH_2_, MDI-β-CD-modified COF, and prepared CSP were characterized by PXRD, FT-IR, SEM and BET. The obtained PXRD spectrum of MDI-β-CD-modified COF (Figure 1a) matched well with the spectra data in the literature [26], illustrating the successful synthesis of MDI-β-CD-modified COF. Furthermore, the diffraction peaks of the COF-TpPa-1 (7.92° and 27.23°) and MDI-β-CD (19.20°) were retained, which proved that the COF-TpPa-1 was modified by the MDI-β-CD. The diffraction peaks of the MDI-β-CD-modified COF and SiO_2_-NH_2_ both appeared in the PXRD spectrum of the MDI-β-CD-modified COF@SiO_2_, which illustrated that the core-shell composite was successfully synthesized.

Comparing the FT-IR spectra (Figure 1b) of the SiO_2_ and SiO_2_-NH_2_, it can be seen that the peak intensity of the Si-OH decreased at 3406 and 974 cm^−1^, whereas the peak intensity of the Si-O-Si enhanced at 1102 cm^−1^, which indicates that there was a condensation reaction between the APTES and the Si-OH. From the FT-IR spectrum of the MDI-β-CD-modified COF in Figure 1b and MDI-β-CD in the Electronic Supplementary Material (Figure S2), the peaks of the MDI-β-CD at 3402 (O-H) and 2897 (-CH-CH-), 2278 (-NCO) and the imine bonds at 1595 and 1644 cm^−1^ (C=N) indicated that the COF-TpPa-1 was modified by the MDI-β-CD. Moreover, the peaks of the MDI-β-CD-modified COF (1595 and 1644 cm^−1^) and SiO_2_-NH_2_ (468, 809, and 1102 cm^−1^) were concurrently displayed in the spectrum of the prepared CSP, which illustrated the successful immobilization of the MDI-β-CD-modified COF on the shell of the SiO_2_-NH_2_. In addition, characteristic peaks at 809, 468, and 1102 cm^−1^ appeared in the spectra of the SiO_2_-NH_2_, SiO_2_ and the prepared core–shell composite, indicating that the growth of the MDI-β-CD-modified COF on the shell of the SiO_2_-NH_2_ did not damage the construction of the spherical silica.

**Figure 2:**
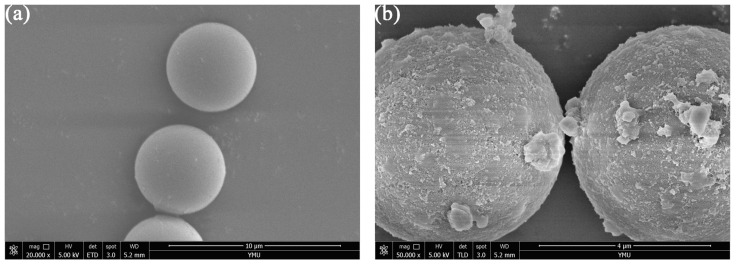
SME images of (**a**) SiO_2_-NH_2_; (**b**) MDI-β-CD-modified COF@SiO_2_.

Figure 2 shows the SEM characterization of the SiO_2_-NH_2_ and core–shell microspheres. The bare SiO_2_-NH_2_ microspheres with particle sizes of ~5 µm have a very smooth surface (Figure 2a). The core–shell microspheres, with a particle size of about 5.3 µm, have a relatively rough surface, which further indicates that the MDI-β-CD-modified COF was effectively grafted on the surface of SiO_2_-NH_2_ microspheres (Figure 2b).

The Brunauer–Emmett–Teller (BET) surface area results of the MDI-β-CD-modified COF have been provided in the Electronic Supplementary Material (ESM) (Figure S1). The BET and pore size distribution (PSD) of the MDI-β-CD-modified COF were measured by N_2_ adsorption/desorption analysis at 77 K. The BET surface area and the total pore volume of MDI-β-CD-modified COF were 43.72 m^2^ g^−1^ and 1.10 cm^3^ g^−1^, respectively. As shown in Figure S1a, the MDI-β-CD-modified COF shows a typical type IV isotherm. The lower BET surface area of the MDI-β-CD-modified COF may be because the MDI-β-CD occupies the pore space of TpPa-1 COF, resulting in insufficient crystallinity and order. The PSD (Figure S1b) of the MDI-β-CD-modified COF shows that the MDI-β-CD-modified COF contains microporous and mesoporous structures.

### 2.2. Resolution of Racemates on the MDI-β-CD-Modified COF@SiO_2_-Packed Column (Column A)

**Figure 3:**
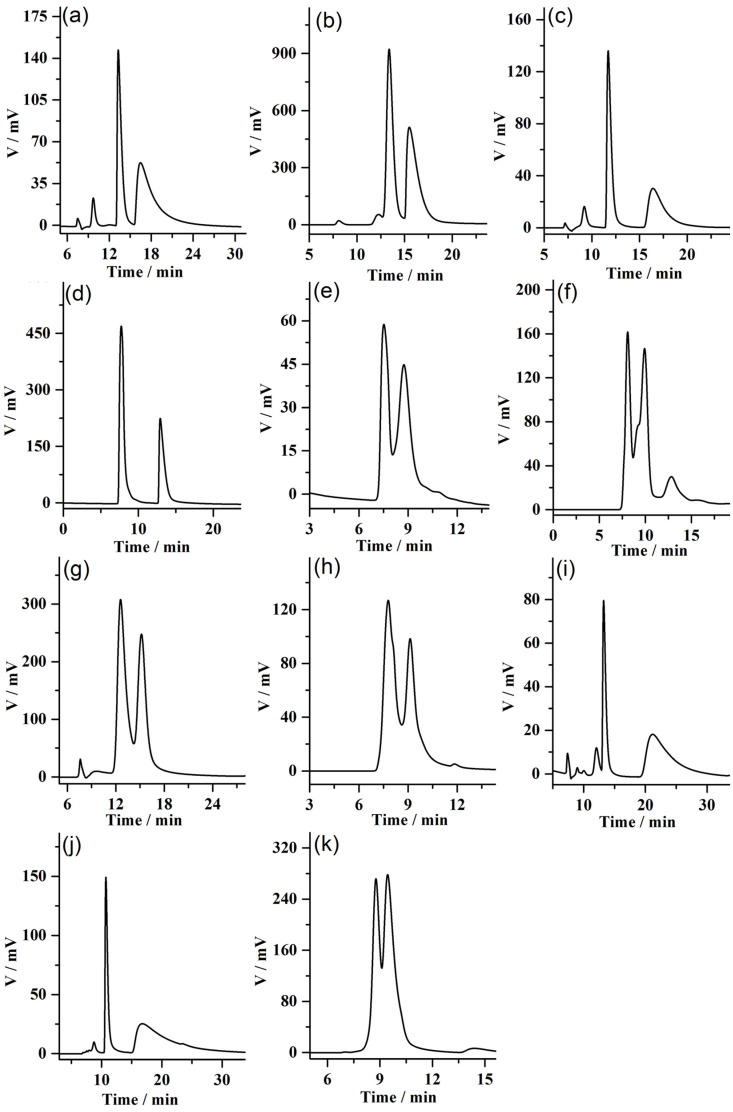
HPLC chromatograms on the MDI-β-CD-modified COF@SiO_2_-packed column (25 cm length × 2.1 mm i.d.) for the separation of racemates: (**a**) (+/−)-hydrobenzoin, (**b**) 1,2-bis(4-fluorophenyl)-2-hydroxyethanone, (**c**) 1-phenyl-1,2-ethanediol, (**d**) trans-stilbene oxide, (**e**) 1-phenylethylamine, (**f**) 2-chloro-2-phenylacetophenone, (**g**) 2,3-dihydro-1H-inden-1-ol, (**h**) benzoin ethyl ether, (**i**) piperoin, (**j**) warfarin, and (**k**) mandelic acid methyl ester. All of racemic compounds were separated at a flow rate of 0.1 mL min^−1^ using a UV detector of 254 nm.

Utilizing n-hexane/isopropanol (90/10, 80/20, 70/30, *v*/*v*) as the mobile phase, the enantioseparation on column A for the racemates was investigated. Eleven pairs of enantiomers were well separated on column A, including (+/−)-hydrobenzoin, 1,2-bis(4-fluorophenyl)-2-hydroxyethanone, 1-phenyl-1,2-ethanediol, trans-stilbene oxide, 1-phenylethylamine, 2-chloro-2-phenylacetophenone, 2,3-dihydro-1H-inden-1-ol, benzoin ethyl ether, piperoin, warfarin, and mandelic acid methyl ester. The structures of these chiral compounds are shown in Figure S3. The chromatographic parameters and HPLC chromatograms of these racemates obtained on column A are shown in Table 1 and Figure 3, respectively.

As shown in Table 1 and Figure 3, four pairs of enantiomers, including 1-phenyl-1,2-ethanediol, trans-stilbene oxide, piperoin, and warfarin, achieved baseline separation on column A. In particular, the highest resolution value for trans-stilbene oxide was as high as 3.26, which indicated that column A had good resolution ability for various racemates. The resolution performance of the fabricated column A was compared with that of the commercial Chiralpak AD-H column and the previously reported β-CD-COF@SiO_2_-packed column (column B) [18]. The comparison results of these racemates separated on the Chiralpak AD-H column and column B are shown in Tables S1 and S2. It can be seen that eight pairs of enantiomers were separated on the Chiralpak AD-H column. Table S1 displays that the resolution values of some enantiomers in the Chiralpak AD-H column were higher than those of them on the column A, and the maximum resolution value of the trans-stilbene oxide reached 5.78. However, three pairs of enantiomers shown in Table S2, (+/−)-hydrobenzoin, 1-phenyl-1,2-ethanediol, and 1-phenylethylamine, were unable to be separated on the Chiralpak AD-H column, but could be separated on column A. From Table S1, more than half of 11 pairs of enantiomers cannot be separated on column B, but can be well separated on the column A. By comparison, the skeleton of MDI-β-CD-modified COF with more benzene rings can provide π-π interaction with analytes containing aromatic rings, which is conducive to the chiral separation process. In addition, column B offered high enantioselectivity for racemates of alcohols, ketones, organic acids and amines, while column A exhibited good separation performance for racemates of alcohols, epoxides, ethers, esters, amines and ketones. The results show that the chiral recognition ability of column A can be complementary to that of Chiralpak AD-H column and column B. 

Column A exhibited good separation ability towards racemates, which was correlated with the chiral microenvironment of the MDI-β-CD-modified COF. The separation mechanism between chiral analytes and CSPs is difficult to explain in detail. From Figure 3, the chromatographic column has the characteristics of long retention time and wide peak for the separation of some chiral compounds, which is attributed to the strong interaction between the second enantiomer and the stationary phase, thus leading to the wide peak to a large extent [27,28]. These racemates, including alcohols, epoxides, ethers, esters, ketones, and amines, can be separated on column A, which may involve multiple interactions such as hydrogen-bonding interactions, π–π interactions, van der Waals forces, dispersion forces, dipole–dipole interactions, and chiral stereo matching to promote the chiral recognition process [18,24]. In particular, some chiral compounds containing hydroxyl groups can be well separated on the column A, such as (+/−)-hydrobenzoin, 1,2-bis(4-fluorophenyl)-2-hydroxyethanone, 1-phenyl-1,2-ethanediol, 2,3-dihydro-1H-inden-1-ol, piperoin, and warfarin. Abundant imine bonds or hydroxyl groups present on the structure of MDI-β-CD-modified COFs easily produce hydrogen-bonding interaction, which is helpful for the resolution of the above-mentioned racemates. Furthermore, COF skeletons containing rich benzene rings easily form π–π interactions with these chrial analytes, which are conducive to the resolution of enantiomers.

### 2.3. Separation of Positional Isomers on the MDI-β-CD-MODIFIED COF@SiO_2_-Packed Column

**Figure 4:**
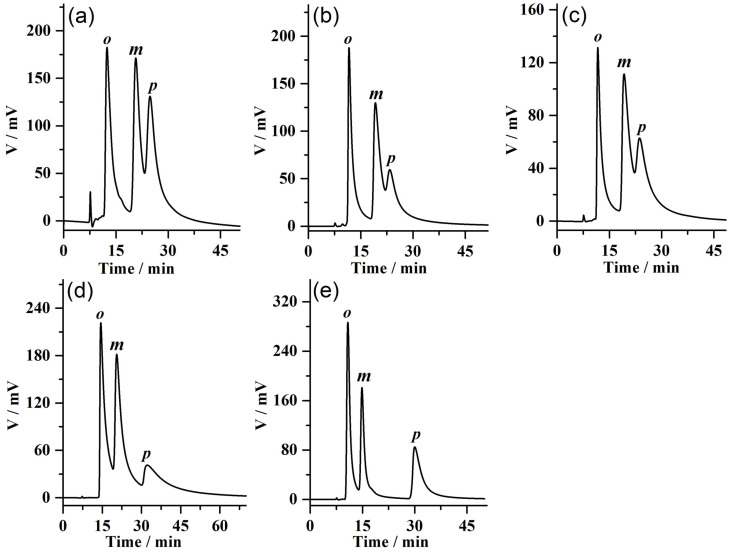
HPLC chromatograms on the MDI-β-CD-modified COF@SiO_2_-packed column (25 cm length × 2.1 mm i.d.) for the separation of positional isomers: (**a**) iodoaniline, (**b**) bromoaniline, (**c**) chloroaniline, (**d**) nitroaniline, and (**e**) dinitrobenzene. All of positional isomers were separated at a flow rate of 0.1 mL min^−1^ and a UV detector of 254 nm.

The separation of positional isomers with similar physicochemical properties is still a challenging issue in the separation. In this study, the chromatographic-separation performance of column A for positional isomers of substituted benzene was investigated. Using n-hexane/isopropanol (90/10, *v*/*v*) as the mobile phase, the column A showed different separation properties for five positional isomers: iodoaniline, bromoaniline, chloroaniline, nitroaniline, and dinitrobenzene. The mobile phase and chromatographic parameters of these positional isomers separated on column A are listed in Table 2. Their chromatograms obtained on this chiral column are presented in Figure 4. The elution order was as follows: p-isomer > m-isomer > o-isomer. It can be seen that all the p-isomers were eluted behind the o-isomers and m-isomers, indicating that the chiral MDI-β-CD-modified COFs had a higher affinity for p-isomers than the other two isomers.

### 2.4. Effect of the Analyte Mass on the HPLC Separation

**Figure 5:**
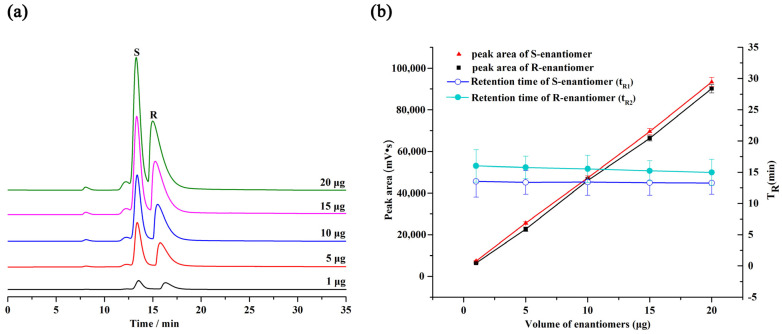
(**a**) HPLC chromatograms of 1,2-bis(4-fluorophenyl)-2-hydroxyethanone at 25 °C on the MDI-β-CD-modified COF@SiO_2_-packed column with different injection masses (1, 5, 10, 15, and 20 μg); (**b**) Effect of injection mass on the retention time and peak area of 1,2-bis(4-fluorophenyl)-2-hydroxyethanone on the MDI-β-CD-modified COF@SiO_2_-packed column containing error bars.

To study the influence of analyte mass on the HPLC separation, we selected 1,2-bis(4-fluorophenyl)-2-hydroxyethanone as the test analyte for separation on the column A with different injection masses at room temperature (Figure 5a). It can be clearly observed in Figure 5b that the peak height and peak area of 1,2-bis(4-fluorophenyl)-2-hydroxyethanone enhanced linearly with the addition of the injected mass, whereas the retention time and selectivity slightly decreased.

### 2.5. Effect of Column Temperature on the HPLC Separation

**Figure 6:**
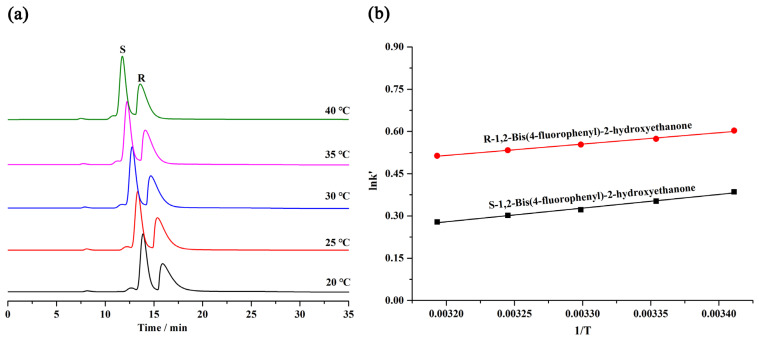
(**a**) HPLC chromatograms of 1,2-bis(4-fluorophenyl)-2-hydroxyethanone on the MDI-β-CD-modified COF@SiO_2_-packed column at different temperatures; (**b**) the van’t Hoff diagram for the separation of 1,2-bis(4-fluorophenyl)-2-hydroxyethanone on the MDI-β-CD-modified COF@SiO_2_-packed column.

To further study the separation thermodynamics on the chromatographic column, 1,2-bis(4-fluorophenyl)-2-hydroxyethanone was selected as the analyte for separation in the temperature range of 20–40 °C. Figure 6a shows that the retention time of the analyte on the column A slowly decreased with the enhancement of the column temperature, confirming that the separation process of the analyte was exothermic. Figure 6b shows that the van’t Hoff plots of the analyte have a good linearity, which demonstrates that the separation mechanism was not affected by the column temperature.

The enthalpy change (ΔH), entropy change (ΔS), and Gibbs-free-energy change (ΔG) were obtained according to the van’t Hoff plots, which are listed in Table 3. The negative numbers of ΔG indicated that the diversion of the analyte from the mobile phase to the CSP was a spontaneous thermodynamic procedure.

### 2.6. Reproducibility and Stability of Column A

**Figure 7:**
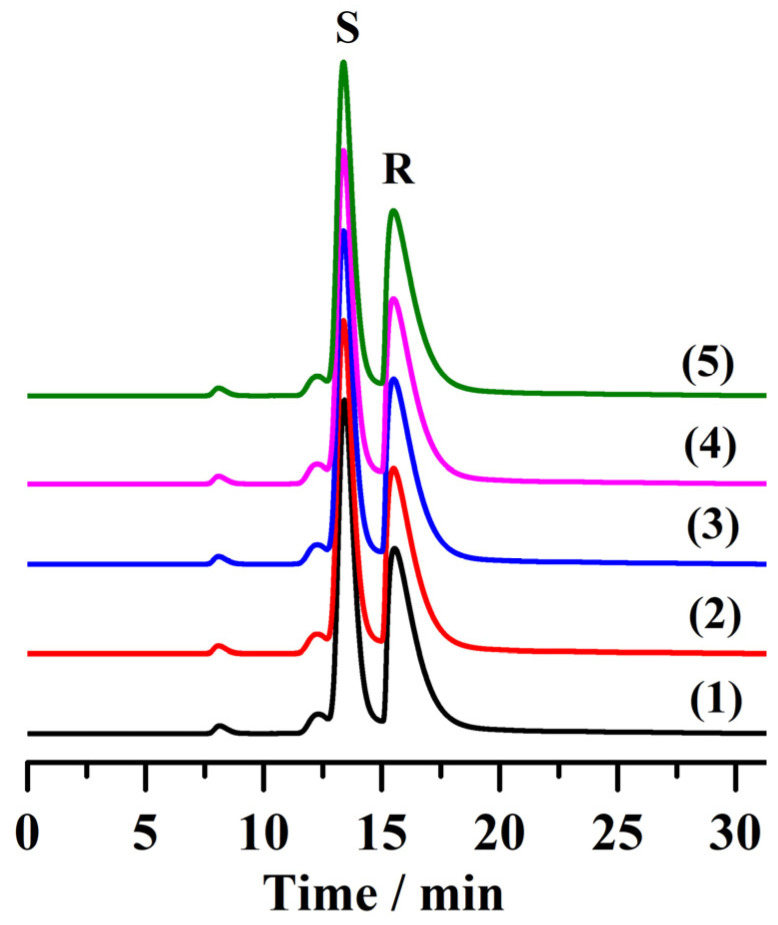
HPLC chromatograms for the separation of 1,2-bis(4-fluorophenyl)-2-hydroxyethanone after repeated injections: (1) 50th injection, (2) 100th injection, (3) 150th injection, (4) 200th injection and (5) 250th injection, respectively. The separation conditions are shown in Table 1 and Figure 3.

To investigate the reproducibility of the chromatographic column A, the 1,2-bis(4-fluorophenyl)-2-hydroxyethanone was selected as the analyte. The chromatograms obtained through repeated injections (50th, 100th, 150th, 200th, and 250th) of the analyte on column A are shown in Figure 7. The retention time and peak area of the analyte remained almost unchanged. The relative standard deviations (RSDs) of the retention time and peak area for the separation of 1,2-bis (4-fluorophenyl)-2-hydroxyethanone (*n* = 5) were 0.28% and 0.79%, respectively, indicating that the chromatographic column had good reproducibility and stability.

## 3. Experimental Section

### 3.1. Chemicals and REAGENTS

The β-Cyclodextrin (β-CD, 99%), 4,4′-methylenebis(phenyl isocyanate) (MID, 95%), 1,4-phenylenediamine (Pa-1, 99%), 1,3,5-trimethylbenzene (95%), 3-aminopropyltriethoxysilane (APTES), and 1,4-dioxane (99%) were obtained from Aladdin Biochemical Technology Co., Ltd. (Shanghai, China). The 2,4,6-Trihydroxybenzene-1,3,5-tricarbaldehyde (Tp, 97%) was bought from Chinese Academy of Sciences-Yanshen Technology Co., Ltd. (Jilin, China). Commercial silica microspheres (5 μm, 120 Å, 300 m^2^/g) were obtained from Nano-Micro Technology Co., Ltd. (Suzhou, China). Ethanol, n-hexane, and isopropanol were bought from Guanghua Technology Co., Ltd. (Guangdong, China). The chiral compounds and positional isomers were bought from Sigma-Aldrich, Adamas-Beta, and TCI.

### 3.2. Instrumentation

The chiral compounds and positional isomers were separated on 230Ⅱ HPLC equipment (Elite Analytical Instruments, Dalian, China). The synthesized core–shell composite was filled into a chromatographic column (250 mm × 2.1 mm i.d.) obtained by Alltech (Dalian, China). The prepared SiO_2_-NH_2_, MDI-β-CD-modified COF, and core–shell composites were characterized by PXRD (Rigaku D/max-3B diffractometer, Tokyo, Japan), FTIR (Nicolet Magna-560 spectrometer, Madison, WI, USA), and SEM (Hitachi Science Systems S-3000N microscope, Tokyo, Japan). Nitrogen adsorption–desorption isotherms of MDI-β-CD-modified COF were measured at 77 K on a Quantachrome EVO (Boynton Beach, FL, USA) instrument.

### 3.3. Synthesis of SiO_2_-NH_2_ Microspheres

Activation of SiO_2_ microspheres: Briefly, spherical silica (5.0 g) were added to a two-neck flask containing 50 mL of 10% HCl solution, and the resulting solution was stirred at 100 °C for 10 h after ultrasonic mixing. Next, the activated SiO_2_ microspheres with a yield of 88% were obtained after centrifugation, washed to neutral with distilled water, and dried under vacuum.

The synthesis of SiO_2_-NH_2_ microspheres was as follows: The activated SiO_2_ (4.0 g) and 3-aminopropyltriethoxysilane (1 mL) were added into a two-necked flask containing 50 mL of anhydrous toluene, and the resulting solution was stirred under N_2_ protection at 80 °C for 24 h. After the reaction, the SiO_2_-NH_2_ microspheres with yield of 80% were collected by centrifugation, repeatedly washed with toluene, acetone and n-hexane, and vacuum-dried at 60 °C for 5 h.

### 3.4. Synthesis of MDI-β-CD-Modified COF

**Figure 8:**
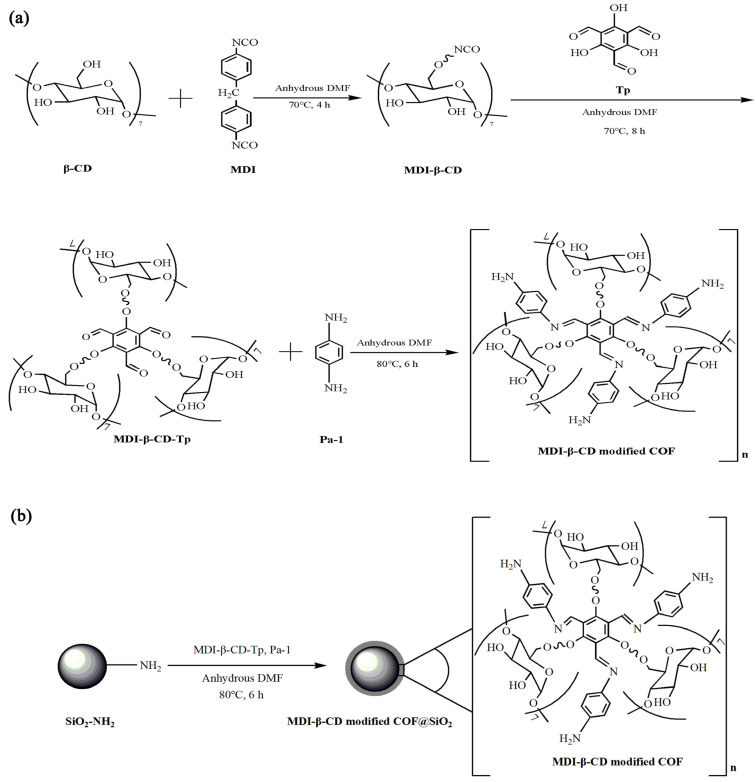
(**a**) Schematic diagram for the synthesis of MDI-β-CD-modified COF; (**b**) Synthesis of the MDI-β-CD-modified COF@SiO_2_ core-shell microspheres.

The fabrication of MDI-β-CD and MDI-β-CD-modified COF were based on previous works [29,30]. First, Tp (0.105 g) and MDI-β-CD (1.066 g) were dispersed in 15 mL of DMF, respectively. The Tp solvent was added into the MDI-β-CD solution under N_2_ protection atmosphere, and the obtained mixed solution was stirred at 70 °C for 8 h. The yellow powder with a yield of 65% was collected after pouring of acetone for precipitation, centrifugation, and vacuum drying. Eventually, MDI-β-CD-Tp (0.431 g) and Pa-1 (0.0541 g) were dispersed in a two-necked flask containing 15 mL of DMF and stirred at 80 °C for 6 h. The powder with a yield of 58% was collected after repeated washing with DMF and ethanol, and vacuum drying for 24 h. The synthesis diagram is shown in Figure 8a.

### 3.5. Synthesis of COF-TpPa-1

The COF-TpPa-1 was synthesized on the basis of a previous study [31]. The Tp (63 mg), Pa-1 (48 mg), mesitylene (1.5 mL), 1,4-dioxane (1.5 mL), and 3 M HAc (0.5 mL) were placed into a beaker. The mixed solution was transferred to a pyrex tube after ultrasonic treatment. The tube was degassed via three freeze–pump–thaw cycles and reacted at 120 °C for 3 days. The collected powder with a yield of 62% was washed with acetone and vacuum-dried for 24 h.

### 3.6. Synthesis of MDI-β-CD-Modified COF@SiO_2_

The preparation process was as follows (Figure 8b): The SiO_2_-NH_2_ (2.0 g), MDI-β-CD-Tp (0.431 g), and Pa-1 (0.0541 g) were added into a two-necked flask (100 mL) containing DMF (15 mL). The mixture reacted at 80 °C for 6 h in N_2_ atmosphere. The yellow product with a yield of 53% was obtained after filtration, repeatedly washed with DMF and ethanol, and vacuum-dried for 24 h.

### 3.7. Column Packing

The chromatographic column was fabricated via a slurry-packing strategy. In short, the prepared CSP (1.2 g) was diffused in n-hexane/isopropanol (90/10, *v*/*v*) solution via ultrasound. Utilizing n-hexane/isopropanol as the substitute solvent, the mixture was quickly poured into a chromatographic column (250 mm × 2.0 mm i.d.) at 25 MPa for 30 min.

### 3.8. Calculation of the Chromatographic Parameters

The fabricated CSP was evaluated by retention factor (k), separation factor (α), resolution values (Rs), Gibbs-free energy (ΔG), enthalpy change (ΔH) and entropy change (ΔS), which were calculated in the light of previous literatures [25,32].

## 4. Conclusions

In conclusion, the chiral MDI-β-CD-modified COF@SiO_2_ core–shell microspheres were synthesized by an in situ growth strategy and utilized as a novel CSP for HPLC separation. The fabricated chromatographic column displayed good separation performance for chiral compounds and positional isomers. The results indicated that the core–shell composite has certain application prospects in the field of chromatographic separation. Nevertheless, it is a challenge to design and synthesize CCOF_S_ and prepare homogeneous COF shell layers on the SiO_2_-NH_2_. Thus, there is still much space for improvement in the fabrication strategy to construct novel chiral COFs, particularly three-dimensional COFs and the optimization of the experiment steps for COF@SiO_2_ materials. We expect that the preparation of COF@SiO_2_ core–shell composites as the CSPs for HPLC chromatographic separation has significant application potential.

## Figures and Tables

**Table 1:** Separation of racemates on the MDI-β-CD-modified COF@SiO_2_-packed column.

Racemates	Mobile Phase n-Hexane/ Isopropanol (*v/v*)	Retention Factor (k)	Separation Factor (α)	Resolution (Rs)
(+/−)-Hydrobenzoin	9:1	1.49	1.44	1.48
1,2-Bis(4-fluorophenyl)-2-hydroxyethanone	9:1	1.51	1.28	1.34
1-Phenyl-1,2-ethanediol	8:2	1.20	1.75	2.17
Trans-stilbene oxide	9:1	0.46	3.15	3.26
1-Phenylethylamine	9:1	0.42	1.55	1.04
2-Chloro-2-phenylacetophenone	9:1	0.52	1.66	0.97
2,3-Dihydro-1H-inden-1-ol	9:1	1.38	1.35	1.14
Benzoin ethyl ether	8:2	0.47	1.54	1.01
Piperoin	9:1	1.50	2.00	2.15
Warfarin	7:3	1.02	2.11	1.60
Mandelic acid methyl ester	7:3	0.65	1.19	0.57

**Table 2:** Separation of positional isomers on the MDI-β-CD-modified COF@SiO_2_-packed column.

Isomers	Mobile Phase n-Hexane/ Isopropanol (*v/v*)	Retention Factor (k)			Separation Factor (α)		Resolution (Rs)	
		m	o	p	α_m-/o-_	α_p-/m-_	R_S1_	R_S2_
Iodoaniline	9:1	1.35	2.90	3.68	4.37	1.27	2.04	0.67
Bromoaniline	9:1	1.20	2.63	3.41	2.19	1.30	2.39	0.72
Chloroaniline	9:1	1.20	2.63	3.47	2.18	1.32	1.98	0.67
Nitroaniline	9:1	1.72	2.87	5.08	1.67	1.77	1.32	1.00
Dinitrobenzene	9:1	1.04	1.80	4.66	1.73	2.59	2.01	4.11

**Table 3:** Values of ΔH, ΔS, ΔG(25 °C), and R^2^ for the R/S-1,2-bis(4-fluorophenyl)-2-hydroxyethanone.

Analytes	ΔH (kJ·mol^−1^)	ΔS (J·mol^−1^·K^−1^)	ΔG (kJ·mol^−1^)	R^2^
S-1,2-bis(4-fluorophenyl)-2-hydroxyethanone	−4.04 ± 0.19	−5.94 ± 0.67	−2.27 ± 0.19	0.9934
R-1,2-Bis(4-fluorophenyl)-2-hydroxyethanone	−3.35 ± 0.13	−1.79 ± 0.42	−2.82 ± 0.13	0.9956

## Data Availability

The data presented in this study are available on request from the corresponding author.

## Supplementary Materials

The following supporting information can be downloaded at: https://www.mdpi.com/article/10.3390/molecules28020662/s1, Figure S1: (a) N_2_ adsorption-desorption isotherms of MDI-β-CD-modified COF; (b) Pore size distribution of MDI-β-CD-modified COF; Figure S2: FT-IR spectra of β-CD, MDI-β-CD, MDI-β-CD-Tp, and COF-TpPa-1; Figure S3: Structures of the chiral compounds separated on the MDI-β-CD-modified COF@SiO_2_-packed column; Table S1: Separation of racemic compounds on the MDI-β-CD-modified COF@SiO_2_-packed column (column A), Chiralpak AD-H column, and β-CD-COF@SiO_2_-packed column (column B); Table S2: Eleven pairs of racemic compounds separated on the MDI-β-CD-modified COF@SiO_2_-packed column and Chiralpak AD-H column (separation conditions as shown in Table S1).

## Sample Availability

Samples of the compounds are not available from the authors.
