# Supplementary material for: Chiral Covalent-Organic Framework MDI-β-CD-Modified COF@SiO2 Core–Shell Composite for HPLC Enantioseparation
Source: Molecules. 2023 Jan 9;28(2):662. doi: 10.3390/molecules28020662 (PMC9866547; doi:10.3390/molecules28020662)
Supplement: Supplementary file 1 [file molecules-28-00662-s001.zip › molecules-2094285-supplementary.pdf]

Supporting Information

Chiral Covalent-Organic Framework MDI-β-CD-Modified COF@SiO₂ Core–Shell Composite for HPLC Enantioseparation

Xiaoyan Ran, Ping Guo, Caifang Liu, Yulan Zhu, Cheng Liu, Bangjin Wang *, Junhui Zhang, Shengming Xie * and Liming Yuan

Department of Chemistry, Yunnan Normal University, Kunming 650500, China
* Correspondence: wangbangjin711@163.com (B.W.); xieshengming_2006@163.com (S.X.)

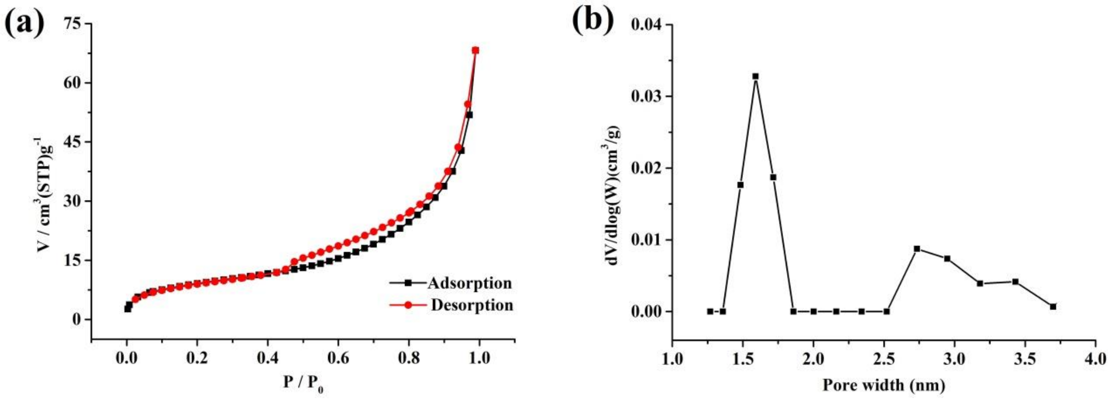

Figure S1. (**a**) N_2 adsorption-desorption isotherms of MDI-β-CD-modified COF; (**b**) Pore size distribution of MDI-β-CD-modified COF

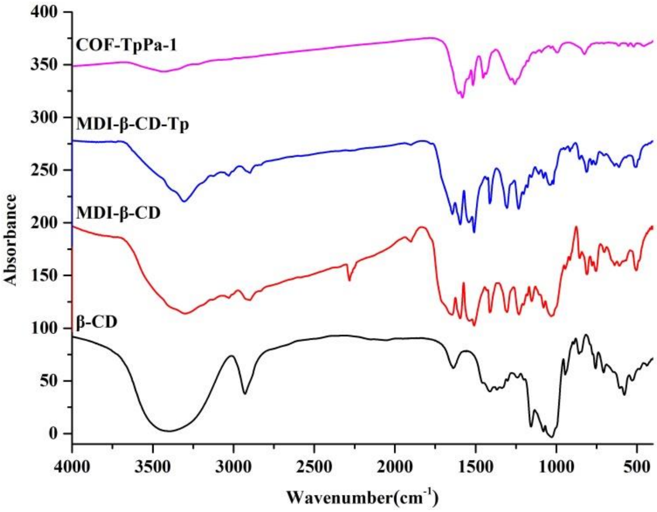

Figure S2. FT-IR spectra of β-CD, MDI-β-CD, MDI-β-CD-Tp, and COF-TpPa-1

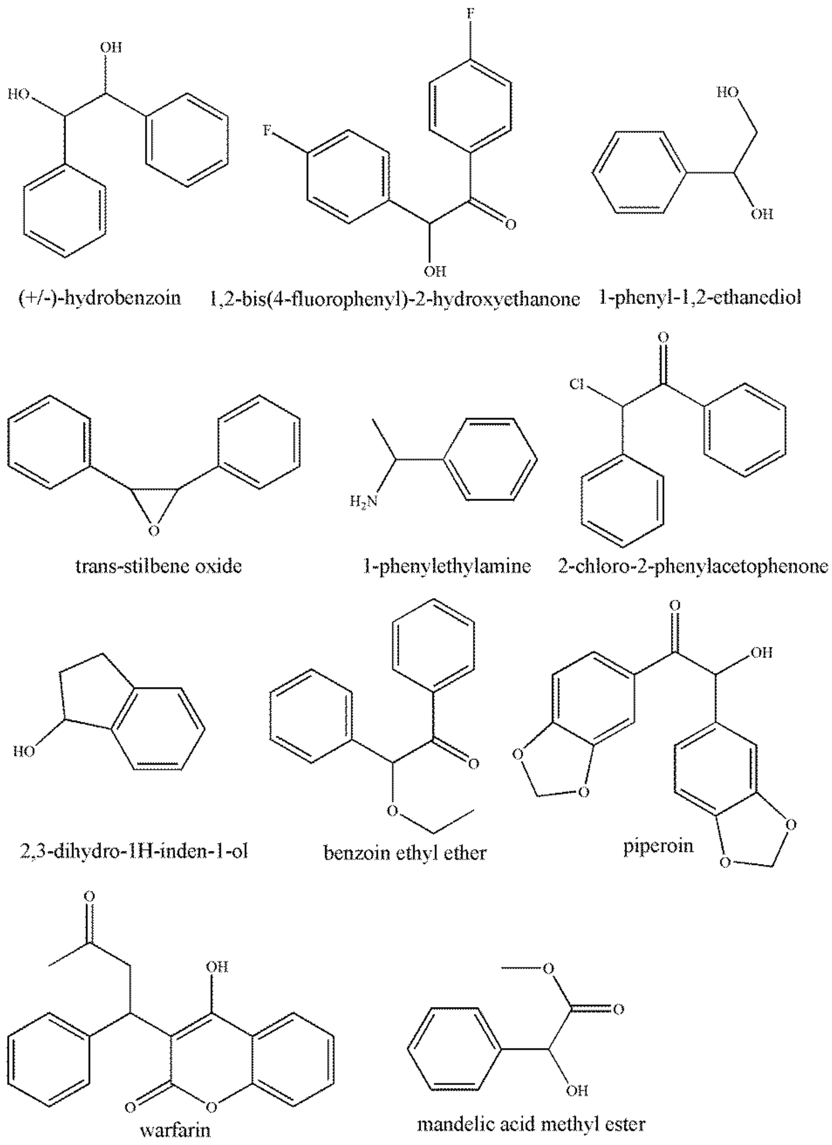

Figure S3. Structures of the chiral compounds separated on the MDI-β-CD-modified COF@SiO_2-packed column

Table S1. Separation of racemic compounds on the MDI-β-CD-modified COF@SiO_2-packed column (column A), Chiralpak AD-H column, and β-CD-COF@SiO_2-packed column (column B)

Racemates	Separation factor(α)			Resolution(Rs)		
	A	AD-H	B	A	AD-H	B
(+/-)-hydrobenzoin[a]	1.44	1.00	2.35	1.48	–[d]	1.77
1,2-bis(4-fluorophenyl)-2-hydroxyethanone[a]	1.28	1.12	1.00	1.34	2.21	–[d]
1-phenyl-1,2-ethanediol[b]	1.75	1.00	1.00	2.17	–[d]	–[d]
trans-stilbene oxide[a]	3.15	3.12	2.37	3.26	5.78	1.65
1-phenylethylamine[a]	1.55	1.00	1.00	1.04	–[d]	–[d]
2-chloro-2-phenylacetophenone[a]	1.66	1.17	1.00	0.97	2.59	–[d]
2,3-dihydro-1H-inden-1-ol[a]	1.35	1.06	1.00	1.14	1.26	–[d]
benzoin ethyl ether[b]	1.54	1.15	1.00	1.01	2.51	–[d]
piperoin[a]	2.00	1.26	1.00	2.15	2.64	–[d]
warfarin[c]	2.11	1.25	1.00	1.60	2.61	–[d]
mandelic acid methyl ester[c]	1.19	1.08	1.00	0.57	1.77	–[d]

Separation conditions: [a]mobile phase, n-hexane/isopropanol (90/10, v/v) as the mobile phase for columns A and AD-H. [b]mobile phase, n-hexane/isopropanol (80/20, v/v) as the mobile phase for columns A and AD-H. [c]mobile phase, n-hexane/isopropanol (70/30, v/v) as the mobile phase for columns A and AD-H; flow rate: 0.1 mL min^{-1}; column temperature: 25 °C. [d]Cannot be separated.

Table S2. Eleven pairs of racemic compounds separated on the MDI-β-CD-modified COF@SiO_2-packed column and Chiralpak AD-H column (separation conditions as shown in Table S1)

**Racemates**	**Chiralpak AD-H**	**MDI-β-CD-modified COF@SiO_2-packed column**
**(+/-)-hydrobenzoin**	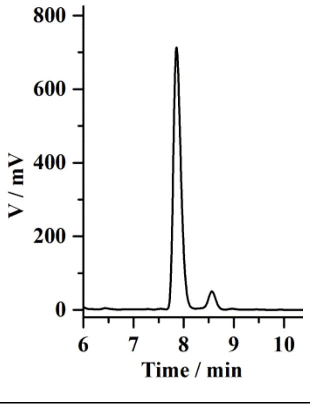	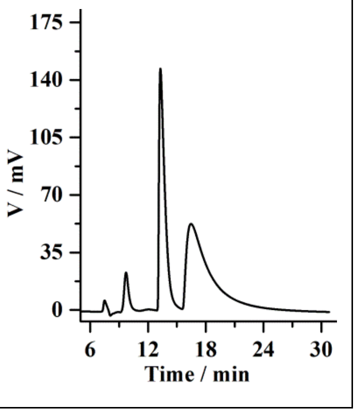

**1,2-bis(4-fluorophenyl)-2-hydroxyethanone**	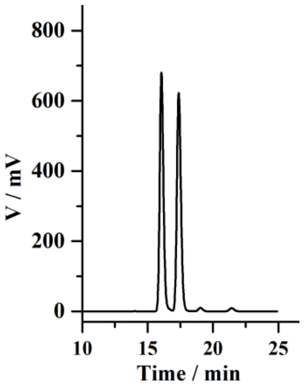	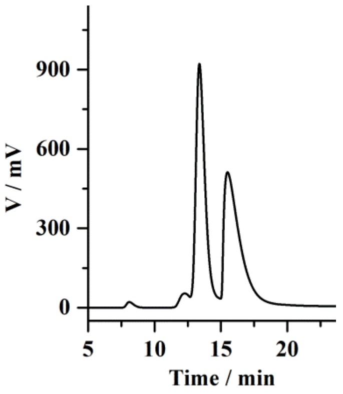
**1-phenyl-1,2-ethanediol**	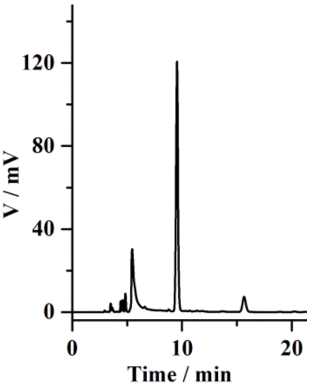	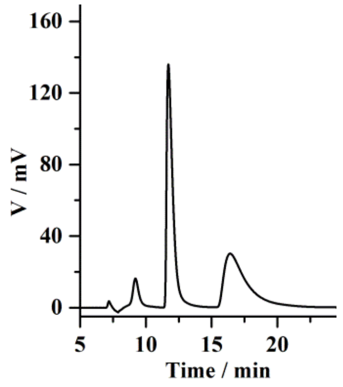
**trans-stilbene oxide**	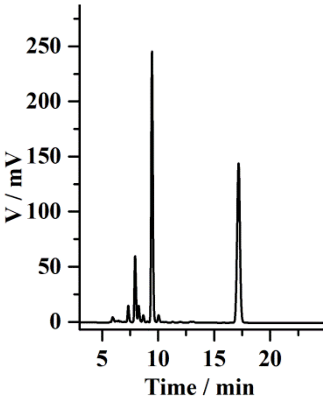	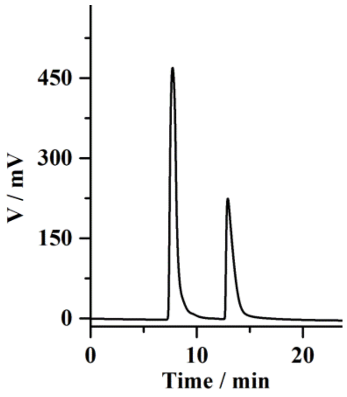
**1-phenylethylamine**	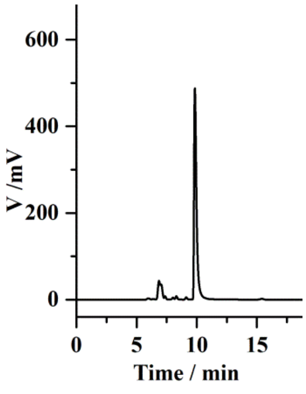	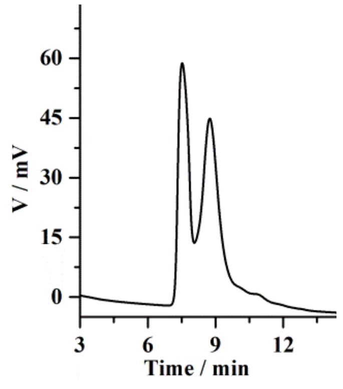

**2-chloro-2-phenylacetophenone**	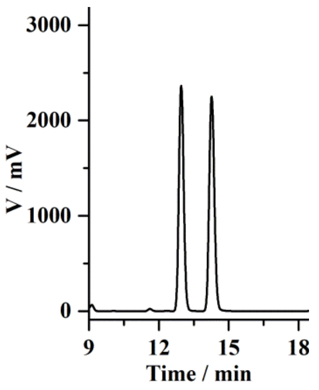	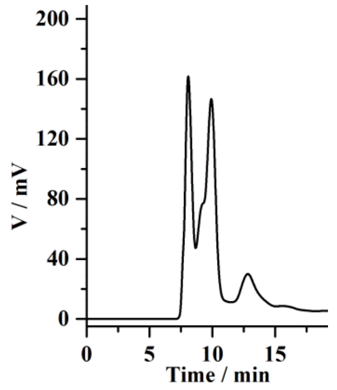
**2,3-dihydro-1H-inden-1-ol**	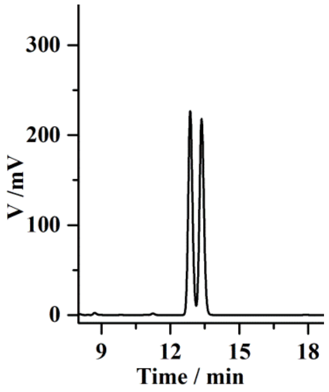	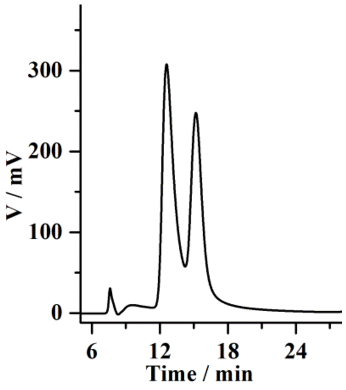
**benzoin ethyl ether**	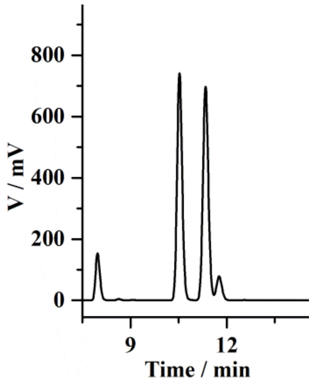	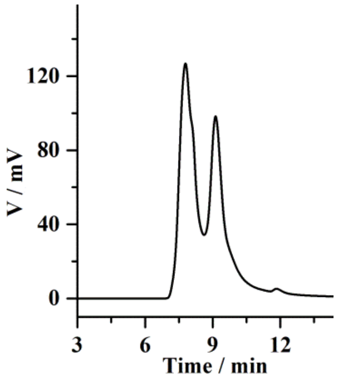
**piperoin**	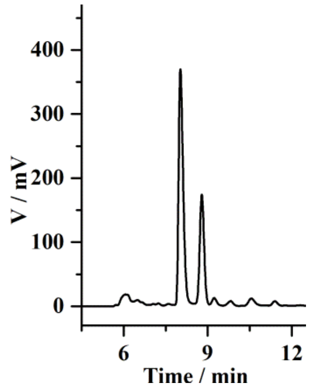	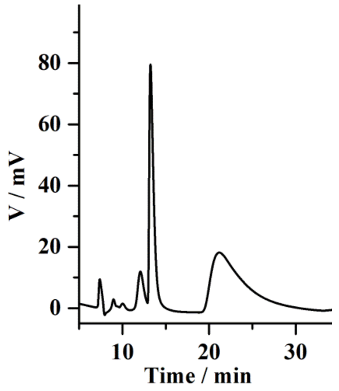

**warfarin**	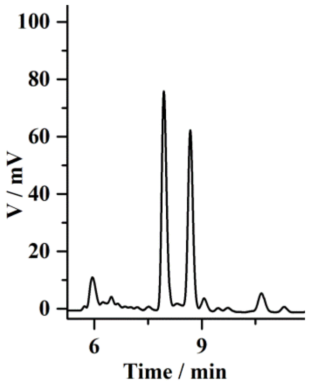	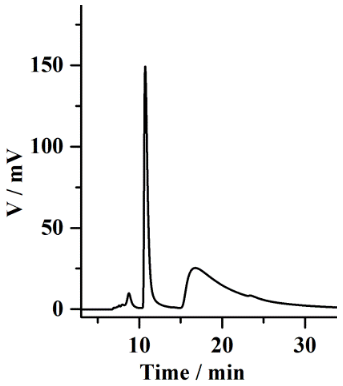
**mandelic acid methyl ester**	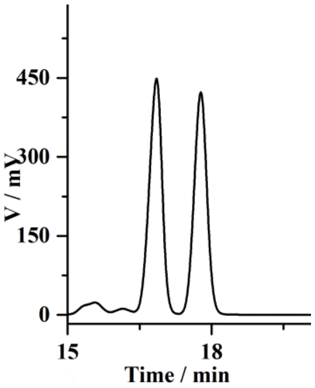	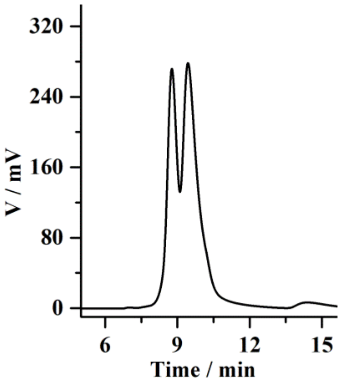
